# The mitochondrial aldehyde dehydrogenase OsALDH2b negatively regulates tapetum degeneration in rice

**DOI:** 10.1093/jxb/eraa045

**Published:** 2020-01-28

**Authors:** Xianrong Xie, Zixu Zhang, Zhe Zhao, Yongyao Xie, Heying Li, Xingliang Ma, Yao-Guang Liu, Letian Chen

**Affiliations:** 1 State Key Laboratory for Conservation and Utilization of Subtropical Agro-Bioresources, Guangzhou, China; 2 Guangdong Laboratory for Lingnan Modern Agriculture, Guangzhou, China; 3 College of Life Sciences, South China Agricultural University, Guangzhou, China; 4 Key Laboratory of Plant Functional Genomics and Biotechnology of Guangdong Provincial Higher Education Institutions, South China Agricultural University, Guangzhou, China; 5 Shanghai Jiao Tong University, China

**Keywords:** Aldehyde dehydrogenase, male sterility, OsALDH2b, programmed cell death, rice, tapetal degeneration

## Abstract

Timely degradation of anther tapetal cells is a prerequisite for normal pollen development in flowering plants. Although several genes involved in tapetum development have been identified, the molecular basis of tapetum degeneration regulation remains poorly understood. In this study, we identified and characterized the nucleus-encoded, conserved mitochondrial aldehyde dehydrogenase OsALDH2b as a key regulator of tapetum degeneration in rice (*Oryza sativa*). *OsALDH2b* was highly expressed in anthers from meiosis to the early microspore stage. Mutation of *OsALDH2b* resulted in excess malonaldehyde accumulation and earlier programmed cell death in the tapetum, leading to premature tapetum degeneration and abnormal microspore development. These results demonstrate that OsALDH2b negatively regulates tapetal programmed cell death and is required for male reproductive development, providing insights into the regulation of tapetum development in plants.

## Teaser

Rice mitochondrial aldehyde dehydrogenase OsALDH2b contributes to tapetal degradation, which is essential for male fertility.

## Introduction

Pollen development is essential to plant reproduction. In rice (*Oryza sativa* L.), which is a staple for over half the global population, abnormal anther development significantly influences rice production (Chen and Liu, 2014). Previous studies have revealed that various regulators such as transcription factors, receptor-like kinases, ATP binding cassette G (ABCG) transporters, glycoproteins, redox homoeostasis-related factors, hormones, and enzymes are involved in anther development and pollen formation in *Arabidopsis* and rice (Wilson and Zhang, 2009; Zhang and Yang, 2014; Zhao *et al.*, 2016; Cai and Zhang, 2018; Yu and Zhang, 2019). The tapetum, the innermost layer in anthers, directly contacts male gametophytes and plays a vital role in microspore development. Tapetum degeneration via programmed cell death (PCD) provides enzymes, sporopollenin precursors, and nutrients for pollen maturation and wall synthesis. Premature or delayed tapetal degradation causes defective pollen development (Ma, 2005). Timely tapetal degradation is strictly controlled by a set of genes including *OsCP1*, *TDR*, *PTC1*, *MTR1*, *EAT1*/*DTD*, *TIP2*, *OsTDF1*, *OsTGA10*, *Cox11*, and *EDT1* (Li *et al.*, 2011; Guo and Liu, 2012; Tan *et al.*, 2012; Ji *et al.*, 2013; Luo *et al.*, 2013; Ko *et al.*, 2014; Zhang and Yang, 2014; Cai *et al.*, 2015; Chen *et al.*, 2018; Bai *et al.*, 2019). Tapetum Degeneration Retardation (TDR), a homolog of Aborted Microspores (AMS) of *Arabidopsis* (Sorensen *et al.*, 2003), interacts with TDR Interacting Protein2 (TIP2) and Eternal Tapetum1 (EAT1) to positively regulate tapetal PCD via two aspartic proteases (AP37 and AP25) and is required for induction of *OsC1* (Li *et al.*, 2006; Niu *et al.*, 2013; Fu *et al.*, 2014). The *tdr* mutant exhibits delayed tapetal PCD and degeneration. However, negative regulators of tapetum degeneration are seldom reported. In addition, recent studies have established that cellular redox status and dynamic reactive oxygen species (ROS) change are essential for tapetal cell specification and punctual initiation of tapetal PCD (Yu and Zhang, 2019; Bai *et al.*, 2019), while glutaredoxin and rice Tapetum Determinant1-Like1A (OsTDL1A)–Multiple Sporocytes1 (MSP1) pathways have a conserved role in determining anther cell fate (Zhang and Yang, 2014; Yu and Zhang, 2019). Rice MADS-box transcription factor3 (OsMADS3) is a key transcriptional regulator acting with the promoter of *OsMT-I-4b* to scavenge ROS during late anther development (Hu *et al.*, 2011). Another ROS-scavenging protein, rice Metallothionein Type 2b (OsMT2b), interacts with Defective Tapetum Cell Death 1 (DTC1) to maintain the normal level of ROS in tapetal cells (Yi *et al.*, 2016).

Reactive metabolites such as ROS and malonaldehyde (MDA) can induce DNA damage (Hu *et al.*, 2011; Zhang and Yang, 2014). Oxidative stress and cellular processes (e.g. lipid peroxidation) produce aldehydes that bear reactive carbonyl groups, which can directly interact with DNA to cause lesions resulting in PCD (Voulgaridou *et al.*, 2011; Biswas and Mano, 2015). Aldehyde dehydrogenases (ALDHs) are a group of NAD(P)^+^-dependent enzymes that catalyse conversion of aldehydes to the corresponding acids. In mammals, ALDHs are commonly detoxifying enzymes that eliminate toxic biogenic and xenobiotic aldehydes (Yoshida *et al.*, 1998; Vasiliou *et al.*, 2000). In plants, most identified ALDHs confer tolerance to abiotic stresses, such as heat, salinity, ultraviolet radiation, and anaerobic conditions (Nakazono *et al.*, 2000; Sunkar *et al.*, 2003; Kotchoni *et al.*, 2006; Rodrigues *et al.*, 2006). Notably, rice OsALDH7 detoxifies aldehydes like MDA and thereby maintains seed viability (Shen *et al.*, 2012; Shin et al., 2009), while OsALDH2a potentially functions in submergence tolerance (Nakazono *et al.*, 2000). A maize (*Zea mays*) gene, *Rf2a*, for restoration of Texas-type cytoplasmic male sterility (CMS-T) encodes a mitochondria-localized aldehyde dehydrogenase (Cui *et al.*, 1996) and also is required for anther development (Liu *et al.*, 2001). However, the details of mitochondrial aldehyde dehydrogenase function in anther development are unclear.

Rice has 22 aldehyde dehydrogenase members grouped into 11 families, including OsALDH7, OsALDH2a, and OsALDH2b (Gao and Han, 2009). In this study, we identified a rice male-sterility mutation caused by a 7-bp deletion in *OsALDH2b*. Furthermore, we revealed that *OsALDH2b* encodes a mitochondrion-targeted aldehyde dehydrogenase enzyme and is highly expressed in anthers during microsporogenesis. Our results demonstrate that OsALDH2b removes excess aldehydes generated during anther development to negatively regulate tapetum degeneration.

## Materials and methods

### Plant materials

All rice plants were grown under natural conditions in South China Agricultural University at Guangzhou’s paddy field. The male-sterile mutant (later named *osaldh2b*) was obtained from a ^60^Co-γ-ray-treated rice Nipponbare (*O. sativa*, ssp. *japonica*) mutant library. The F_2_ mapping population was generated from a cross between the mutant and an *indica* variety, Huanghuazhan (HHZ, *O. sativa*, ssp, *indica*). In the F_2_ population, male-sterile plants were selected primarily for genetic mapping. For screening recombinant individuals, F_2_ and F_3_ segregants were planted in 96-well plates and used for high-throughput DNA preparation as described previously (Wang *et al.*, 2013).

### Mutant phenotype characterization

Plants were photographed with a Nikon digital camera. Flowers were photographed with a stereomicroscope (SZx10/DP72, Olympus, Japan). Pollen grains were stained with 1% I_2_–KI solution and photographed with a ﬂuorescence microscope (Axio Observer Z1, Zeiss, Germany). Preparation of rice anther sections for light microscopy and electron microscopy was performed as previously described (Li *et al.*, 2006).

### Map-based cloning of *OsALDH2b*

A set of 145 male-sterile plants segregated from 652 F_2_ individuals was used for primary mapping. Recombinants were then screened from F_2_ and F_3_ families for fine mapping with newly developed insertion/deletion (InDel) molecular markers (see Supplementary Table S1 at *JXB* online). Rice genomic DNA samples were prepared from fresh leaf tissues using 1% sodium dodecyl sulfate.

### Vector construction for transgenic plants

For the functional complementation test, a 12.2 kb wild-type genomic fragment of *OsALDH2b* was amplified by three steps. The first fragment was amplified using OsALDH2b-F1 and OsALDH2b-R1 primers (Supplementary Table S2) and cloned into the *Mlu*I and *Sal*I sites of the binary vector pCAMBIA1300.2. The second fragment was amplified with OsALDH2b-T5F2 and OsALDH2b-T5R2 primers and cloned, into the positive clones produced in the first step, at the *Sal*I site using an isothermal *in vitro* recombination (IR) system (Jiang *et al.*, 2013). The third fragment, containing the 5′-upstream region, was amplified with pALDH2b-T5F and pALDH2b-T5R primers, and inserted into the vector constructed in step 2 at the *Bam*HI site by the IR method. The CRISPR/Cas9 genome-targeting construct for *OsALDH2b* (target site: TGGGACACAAGGATTGTTGC*CGG*; protospacer adjacent motif italicized) was designed with the web-based CRISPR-GE toolkit (http://skl.scau.edu.cn/) (Xie *et al.*, 2017) and prepared using the CRISPR/Cas9 vector system (Ma *et al.*, 2015). All constructs were introduced into rice with *Agrobacterium*-mediated transformation. Positive transformants were screened with *HPT* primers by PCR. The target site sequences of gene knockout mutants were sequenced and decoded with CRISPR-GE/DSDecodeM (Liu *et al.*, 2015; Xie *et al.*, 2017).

### RNA extraction and qRT-PCR

Total RNA was extracted from rice tissues using TRIZOL reagent (Thermo Fisher Scientific, USA), and isolated RNA was treated with DNase I. The treated RNA was then used for first-strand cDNA synthesis with oligo (dT) using the first-strand cDNA synthesis kit (Promega, USA). Two microliters of the reverse transcription product was used as the template for PCR reactions. The quantitative reverse transcription polymerase chain reaction (qRT-PCR) of *OsALDH2b* and other genes related to anther development used the primers listed in Supplementary Table S2.

### Subcellular localization

The coding region of *OsALDH2b* was amplified from wild-type cDNA with the primers OsALDH2b-cF and OsALDH2b-cR (Supplementary Table S2). After digestion with *Hin*dIII and *Bam*HI, the fragments were fused in-frame with the enhanced green fluorescent protein (eGFP) coding sequence (Heim *et al.*, 1995; Cormack *et al.*, 1996), subcloned into a pUC-18-based vector and driven by the CaMV35S promoter to produce the transient expression vector ALDH–eGFP. A mutant orange fluorescent protein (mOrange) fused with a mitochondrial transit signal peptide derived from RF1b (Wang *et al.*, 2006) was prepared as a positive control (RF1b–mOrange). These constructs were bombarded into onion epidermal cells by a helium-driven accelerator (PDS/1000; Bio-Rad, USA). Cells that exhibited eGFP and mOrange fluorescence were imaged with a laser scanning confocal microscope (LSM7 DUO, Zeiss, Germany).

### Aldehyde dehydrogenase enzymatic assays

Full-length *OsALDH2b* cDNA (excluding the mitochondria-targeted sequence) was isolated with the primers OsALDH2b-cFD and OsALDH2b-cR (Supplementary Table S2) and cloned into the pET32a(+) vector fused with a His-tag. The resultant plasmid was transformed into *E. coli* strain BL21 (DE3). Once the OD_600_ reached approximately 0.6, transformed cells were incubated at 18 °C for 16 h with 1 mM isopropylthio-β-galactoside. The supernatant containing extracted proteins was purified with a Ni–nitrilotriacetic acid spin column. For the enzymatic assay, aldehyde was the substrate. ALDH enzymatic activity for reduction of NAD^+^ to NADH was evaluated by the increase of absorbance at 340 nm (Shin *et al*., 2009).

### Measurement of malonaldehyde content in the anthers

Determination of MDA levels was by the thiobarbituric acid (TBA) method (Loreto and Velikova, 2001). Anther samples at different developmental stages (each 100 mg) were homogenized in 2 ml of 0.1% trichloroacetic acid solution, and the extract was centrifuged at 12 000 *g* for 15 min; 0.5 ml of the supernatant was diluted to 1 ml with 0.5% TBA in 20% trichloroacetic acid. The mixture was heated at 95℃ for 30 min and then cooled on ice. Supernatant absorbance was measured at 530 nm with a Synergy Mx Multi-Mode Reader (BioTek, USA), subtracting non-specific absorbance at 600 nm.

### TUNEL assay

Anther developmental stages were confirmed by observing anther cross-sections with light microscopy. Preparation of anther sections and a terminal deoxynucleotidyl transferase-mediated dUTP nick-end labeling (TUNEL) assay used a Dead End Fluorometric TUNEL Kit (Promega, USA); these were performed as previously described (Li *et al.*, 2006; Luo *et al.*, 2013). Fluorescein’s green fluorescence (TUNEL signal) and propidium iodide’s red fluorescence were imaged with 488 nm (excitation) and 520 nm (detection), and 488 nm (excitation) and 610 nm (detection), respectively, under a LSM 7 DUO laser scanning confocal microscope (Zeiss, Germany).

## Results

### Identification and phenotype of the *osaldh2b* mutant

**Fig. 1.**
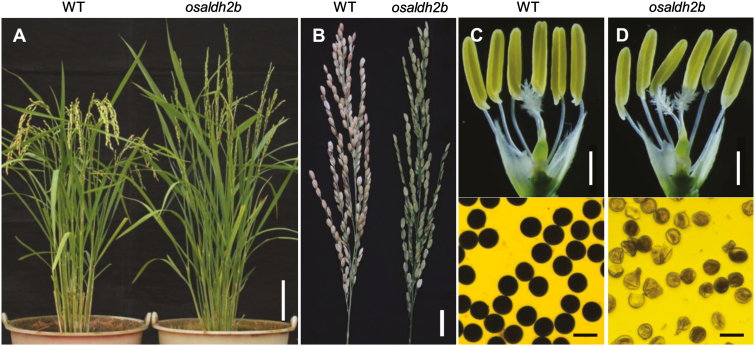
Phenotypic comparison between wild-type (WT) and *osaldh2b*. (A) WT plants (left) and *osaldh2b* (right) at the heading stage. Scale bar: 10 cm. (B) Comparison of seed setting between WT (left) and *osaldh2b* (right). Scale bar: 2 cm. (C) Spikelet (top) and pollen grains (bottom) of WT. (D) Spikelet (top) and pollen grains (bottom) of *osaldh2b*. Pollen grains were stained with I_2_–KI. Scale bar: 1 mm for spikelet and 50 μm for pollen grains.

We obtained a male-sterile mutant by screening a rice mutant library (*japonica* cultivar Nipponbare) created with ^60^Co-γ-ray radiation. The mutant exhibited normal vegetative and panicle development, but failed to generate viable pollen, and never set seed (Fig. 1A–D). Analysis revealed that the sterility was caused by a loss-of-function mutation located in the *OsALDH2b* gene (see below); we therefore named this mutant *osaldh2b*. We crossed mutant plants with pollen grains from the *indica* variety (HHZ) to generate F_1_ hybrids. We examined 652 F_2_ individual plants that resulted from the cross, and observed that 507 plants were male fertile and 145 male sterile (χ ^2^=2.65 for 3:1, *P*>0.05) (Supplementary Table S3), indicating that the male-sterility phenotype was controlled by a recessive locus.

### Map-based cloning and functional validation of *OsALDH2b*

**Fig. 2.**
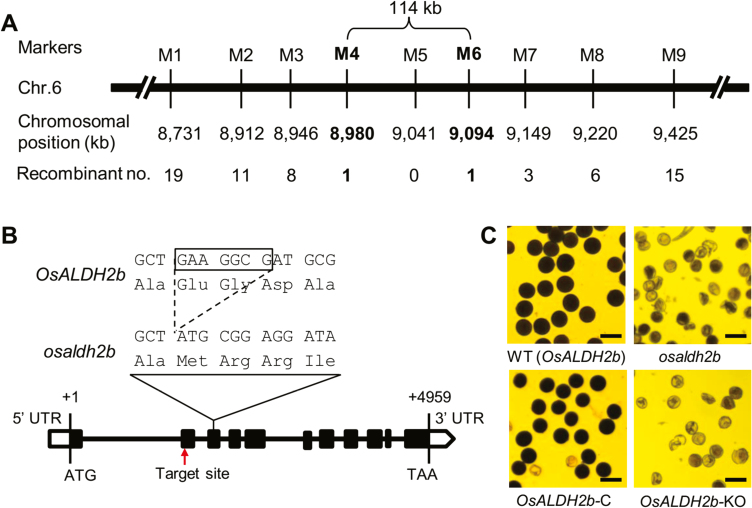
Molecular identification and functional validation of *OsALDH2b*. (A) The mutant gene was mapped within a 114-kb region on chromosome 6, using 6652 total segregants from the F_2_ and F_3_ families. The molecular markers (M1–M9) and physical positions on the chromosome are indicated. (B) Diagram showing *OsALDH2b*’s genomic structure within the mapped region. A 7-bp deletion (boxed) occurred in the third exon of *osaldh2b*. The start codon (ATG) and the stop codon (TAA) are indicated. Red arrow indicates the editing site targeted with CRISPR/Cas9. (C) Pollen phenotypes of wild-type (WT, fertile; top left), the mutant (*osaldh2b*, sterile; top right), the functionally complemented mutant plant with transgenic *OsALDH2b* (*OsALDH2b-*C, fertile; bottom left), and the *OsALDH2b-*knockout plant (*OsALDH2b-*KO, sterile; bottom right). Scale bars: 50 μm.

To isolate the mutated gene conferring male sterility, we used 6652 segregants from the F_2_ and F_3_ families and a set of polymorphic markers covering the entire genome, and mapped the mutant locus to a 114-kb region on chromosome 6 (Fig. 2A). DNA sequencing analysis in this region of the mutant revealed a 7-bp deletion in the third exon of *OsALDH2b* (LOC_Os06g15990, annotated by RGAP7; or Os06g0270900, annotated by RAP-DB), causing a frame shift to introduce a premature stop codon at the 125th amino acid (Fig. 2B).

To confirm that the male-sterile phenotype resulted from the mutation in *OsALDH2b*, we prepared a binary construct (*OsALDH2b*-C) that carried a 12.2-kb genomic DNA fragment of wild-type *OsALDH2b* to thoroughly test functional complementation of this fragment comprising *OsALDH2b*’s 4.2-kb upstream 5′-UTR/promoter sequence, the entire 5.4-kb coding region (including introns), and a 2.6-kb downstream region. We transformed this construct into calli induced from heterozygous *OsALDH2b*/*osaldh2b* plants. Of 26 *OsALDH2b*-C transgenic plants (T_0_), six plants were homozygous for the *osaldh2b* allele, and these exhibited normal male fertility (Fig. 2C). Next, we used CRISPR/Cas9-based genome editing to knock out *OsALDH2b* in wild-type plants. As expected, the *OsALDH2b-*knockout plants (*OsALDH2b*-KO) exhibited a male-sterile phenotype, similar to the *osaldh2b* mutant (Fig. 2C; Supplementary Fig. S1). Therefore, we concluded that *OsALDH2b* is required for male development in rice.

### OsALDH2b and its orthologs are highly conserved in monocot and eudicot species

**Fig. 3.**
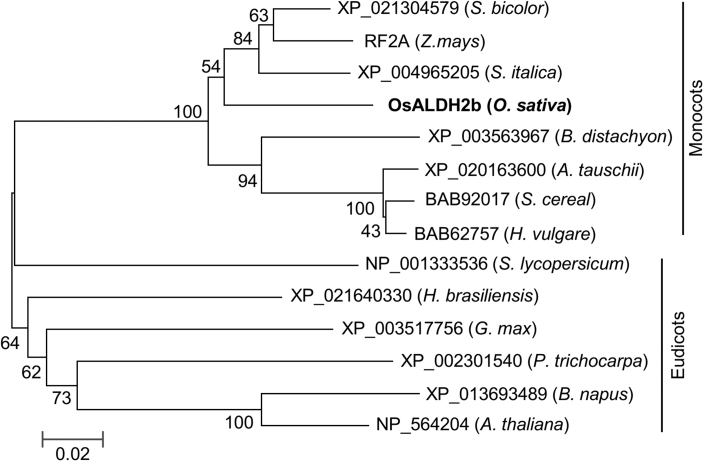
Phylogenetic analysis of ALDH orthologs in representative plants. A phylogenetic tree for ALDHs of eudicot and monocot plants was built with the neighbor-joining method (bootstrap test, 1000 replicates) with the full-length protein sequences.

To gain insight into OsALDH2b’s evolutionary history, we used BLASTP with OsALDH2b’s full-length amino acid sequence to find orthologs and create a phylogenetic tree. We retrieved 13 orthologous sequences from seven monocot species and six eudicot species from the database. Comparing these proteins showed that OsALDH2b had the highest similarity to orthologs from monocot plants (*Sorghum bicolor*, 91%; *Zea mays*, 92%; *Setaria italic*, 91%; *Brachypodium distachyon*, 90%; *Hordeum vulgare*, 88%; *Secale cereal*, 88%; and *Aegilops tauschii*, 88%). Similarity to eudicot orthologs was lower (*Arabidopsis*, 81%; *Solanum lycopersicum*, 79%; *Hevea brasiliensis*, 79%; *Glycine max*, 78%; *Populus trichocarpa*, 77%; and *Brassica napus*, 76%) (Supplementary Fig. S2). A phylogenetic tree generated using MEGA7 (Kumar *et al.*, 2016) divided the proteins into two groups, monocots and eudicots (Fig. 3). Of the orthologs we identified, the maize protein (RF2A) is required for anther development and is a functionally characterized fertility restorer for CMS-T maize lines (Liu *et al.*, 2001; Cui *et al.*, 1996). These data suggest that the ALDH-like proteins are highly conserved among monocot and eudicot plants, and potentially share a conserved function in male reproductive development.

### *OsALDH2b* encodes a mitochondrial aldehyde dehydrogenase and is highly expressed in anthers

**Fig. 4.**
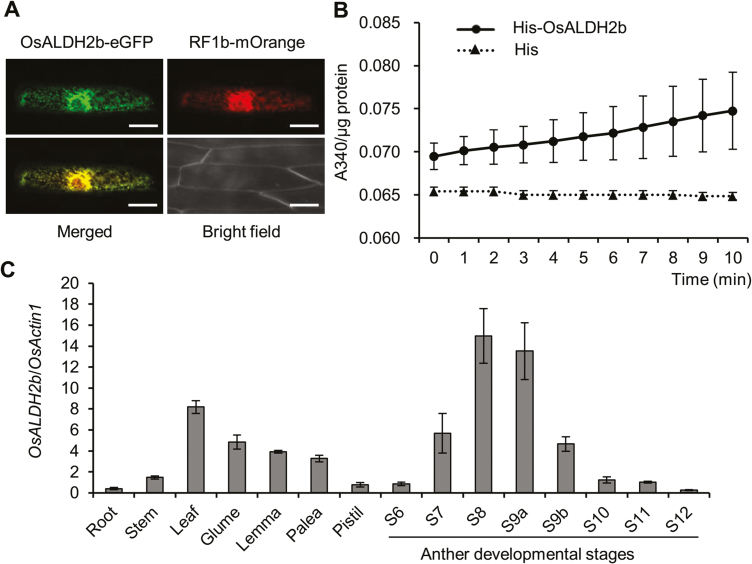
Characterization of OsALDH2b. (A) Subcellular localization of OsALDH2b. p35S::OsALDH2b-eGFP and p35S::RF1b-mOrange vectors were co-bombarded into onion epidermal cells. Scale bars: 50 μm. (B) *In vitro* OsALDH2b enzymatic assay. (C) *OsALDH2b* expression pattern. The *OsActin1* gene was used for normalization. Data are shown as means ± SD (*n*=3).

Sequence analysis showed that *OsALDH2b* encodes a predicted 549 amino acid mitochondrial aldehyde dehydrogenase (Supplementary Fig. S3). To verify OsALDH2b’s subcellular localization, we co-transformed OsALDH2b–eGFP and an RF1b–mOrange control into onion epidermal cells. Images demonstrated that the OsALDH2b–eGFP signal co-localized with RF1b–mOrange in mitochondria, indicating that OsALDH2b is a mitochondrion-localized protein (Fig. 4A).

To examine whether OsALDH2b has enzymatic activity for aldehydes, we purified recombinant OsALDH2b–His to use in enzyme activity assays. Here, reduction of NAD^+^ to NADH was measured as the increase in absorbance values at *A*_340_. When aldehyde was used as the substrate, recombinant OsALDH2b–His exhibited significant enzymatic activity (Fig. 4B), demonstrating that the protein has aldehyde dehydrogenase activity.

To investigate *OsALDH2b* function, we analysed its expression pattern during rice development with qRT-PCR. *OsALDH2b* was expressed in both vegetative and reproductive organs. During anther development, *OsALDH2b* was highly expressed from the meiosis I stage (S7) until the middle microspore stage (S9b), and peaked at the meiosis II/tetrad (S8) and early microspore (S9a) stages (Fig. 4C); rice anther stages (S1–S12) were assigned as previously described (Zhang *et al.*, 2011). *OsALDH2b*’s expression profile was consistent with transcriptome data (RiceXPro, http://ricexpro.dna.affrc.go.jp/) (Supplementary Fig. S4). These data suggest that *OsALDH2b* may function in anther and pollen development from meiosis to the microspore stages.

### The *osaldh2b* mutant anthers accumulate excess malonaldehyde

Cellular redox state is a key factor for male gametogenesis (Zhang and Yang, 2014). MDA is the predominant product of oxidative stress and one of the most highly reactive of the endogenous aldehydes, which are triggered by hypoxic status during early anther development and potentially produce toxic byproducts (Voulgaridou *et al.*, 2011). To determine whether OsALDH2b acts in anther development by reducing the aldehyde accumulation, we used a TBA assay to measure MDA content in anthers of both wild-type and *osaldh2b* plants.

**Fig. 5.**
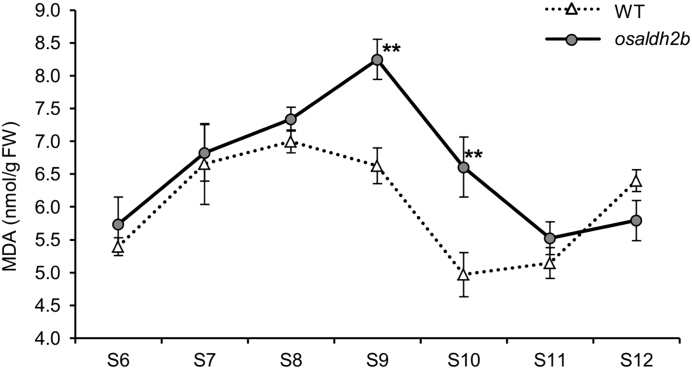
MDA content dynamics during anther development. MDA content of *osaldh2b* anthers was significantly higher than that in WT anthers at microspore stages (S9 and S10). These data are derived from three replicates; **P<0.01 by *t*-test.

In wild-type anthers, MDA content gradually increased from the microspore mother cell stage (S6) to meiosis/tetrad stages (S7/S8), and then decreased until the late microspore stage (S10) (Fig. 5). In *osaldh2b* anthers, however, the MDA level was much higher from the S8 to S10 stages. This result indicated that OsALDH2b acts as a detoxifying enzyme that eliminates aldehydes generated during anther and microspore development.

### The *osaldh2b* mutant exhibits premature tapetal programmed cell death

**Fig. 6.**
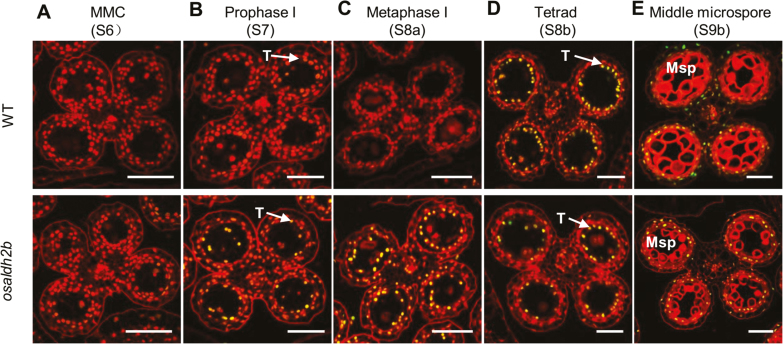
Tapetal nuclear DNA fragmentation in WT and *osaldh2b* anthers. The anthers in WT (top) and *osaldh2b* (bottom) from the microspore mother cell stage through the middle microspore stage were compared for nuclear DNA fragmentation (indicating PCD) using the TUNEL assay (A–E). Nuclei were stained with propidium iodide (red fluorescence); yellow signals indicate TUNEL-positive nucleus staining. MMC, microspore mother cell; T, tapetum; Msp, microspore. Scale bars: 50 µm.

MDA is a highly reactive aldehyde that reacts strongly with DNA and proteins (Voulgaridou *et al.*, 2011). To examine whether MDA accumulation affects DNA fragmentation in *osaldh2b* anthers, we performed a TUNEL assay on anthers across developmental stages. Wild-type anthers showed strong TUNEL-positive signals in tapetal cells at the tetrad stage (S8b) (Fig. 6A–E, top). However, in *osaldh2b* tapetal cells, we detected TUNEL-positive signals in earlier stages, particularly in the metaphase I stage (S8a) (Fig. 6A–E, bottom). These results demonstrate that PCD-induced tapetal DNA fragmentation occurred at an earlier time point in the *osaldh2b* mutant, suggesting that excess MDA accumulation in the mutant’s developing anthers may accelerate PCD in tapetum cells.

### The *osaldh2b* mutant exhibits abnormal tapetal degeneration and microspore development

**Fig. 7.**
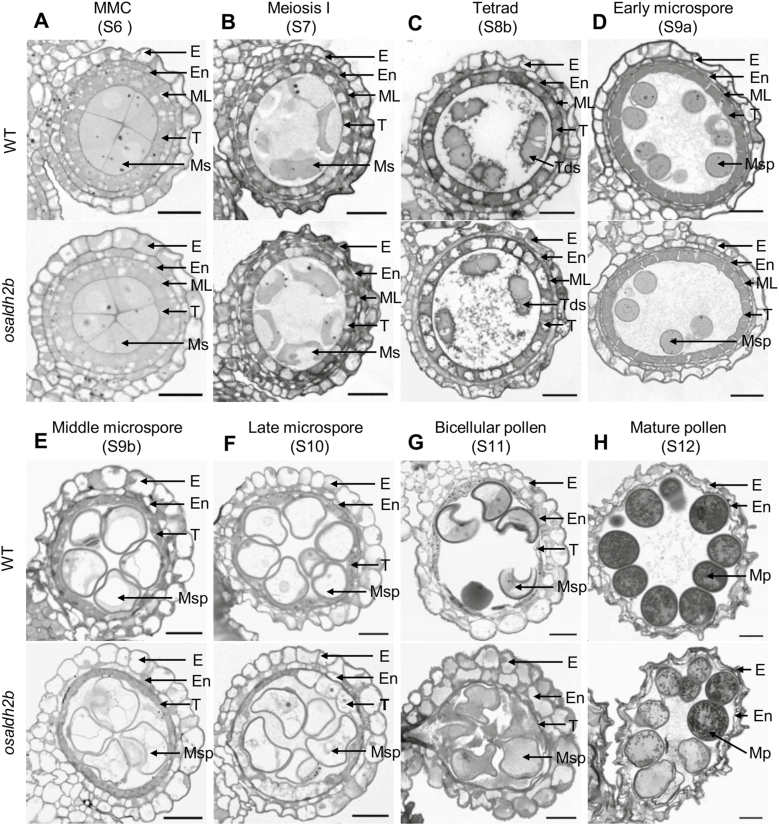
Transverse section analysis reveals anther development in WT and *osaldh2b*. (A–D) No obvious differences were observed between WT (top) and *osaldh2b* (bottom) anthers from S6 to S9a. (E) Compared with WT anthers, *osaldh2b* anthers displayed thinner tapetum and irregular microspores in the locule at S9b. (F) The *osaldh2b* tapetum was less condensed and weakly stained, and microspores were collapsed with uneven cytoplasm at S10. (G) The *osaldh2b* anther wall layers, including epidermis and endothecium, appeared disordered, enlarged, and broken, and exhibited severely abnormal microspores at S11. (H) The *osaldh2b* anthers at S12 exhibited collapsed pollen grains with no or less cellular content accumulation. E, epidermis; En, endothecium; ML, middle layer; Mp, mature pollen; Ms, microsporocyte; Msp, microspores; T, tapetum; Tds, tetrads. Scale bars: 20 μm.

To further investigate the role of OsALDH2b during male reproductive development, we analysed semi-thin sections of wild-type and mutant anthers. We observed no obvious differences between cells in wild-type and *osaldh2b* at early developmental stages (microspore mother cell stage to the early microspore stage) (Fig. 7A–D). In both wild-type and *osaldh2b* anthers, microsporocytes and somatic layers (including the epidermis, endothecium, middle layer, and tapetum) exhibited characteristic structures (Fig. 7A). Microsporocytes in both wild-type and *osaldh2b* had progressed through normal meiosis (S7), during which the tapetum had become vacuolated (Fig. 7B); subsequently, tetrads of haploid microspores had formed (S8) (Fig. 7C). At the early microspore stage (S9a) in both genotypes, free microspores had been released from tetrads, the middle layer appeared thin, and the tapetum looked condensed, less vacuolated, and deeply stained (Fig. 7D).

We detected morphological differences between *osaldh2b* and wild-type anthers starting from the middle microspore stage (S9b): the wild-type tapetum was evident and microspores were round and vacuolated (Fig. 7E, top). The *osaldh2b* tapetum, however, appeared thinner, and microspores appeared irregularly shaped (Fig. 7E, bottom). At the late microspore stage (S10), wild-type tapetum had become hill-shaped, appeared highly condensed and deeply stained, and formed microspores containing a single, large central vacuole (Fig. 7F, top). By contrast, *osaldh2b* tapetum was less condensed and weakly stained; microspores appeared collapsed and exhibited uneven cytoplasm associated with abnormal vacuolization (Fig. 7F, bottom). At the bicellular pollen stage (S11), wild-type anthers exhibited typical falcate-shaped pollen grains and completely degenerated tapetal cells (Fig. 7G, top). At this stage, *osaldh2b* anther wall layers, including the epidermis and endothecium, appeared disordered, enlarged, and broken; mutant plants had produced severely aberrant microspores (Fig. 7G, bottom). At the mature pollen stage (S12), in contrast to wild-type, *osaldh2b* pollen grains were irregularly shaped and had accumulated no or less storage materials, and the anthers had shriveled (Fig. 7H).

**Fig. 8.**
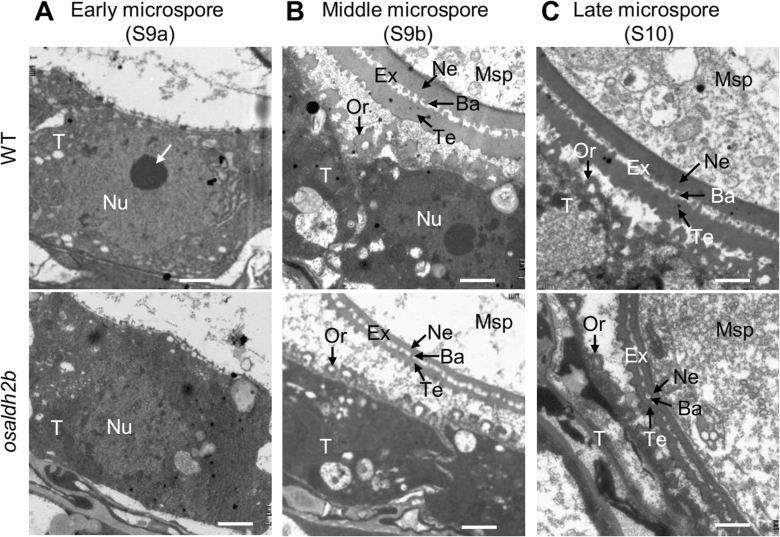
Transmission electron micrographs of WT and *osaldh2b* anthers. Tapetum and microspores in WT (top) and *osaldh2b* (bottom) anthers from S9a to S10 are shown. (A) In WT tapetum at S9a, nuclei appeared intact with a visible nucleolus (arrowed); the nucleolus had disappeared in the *osaldh2b* tapetum at this stage. (B) At S9b, nuclei in *osaldh2b* tapetal cells were completely degenerated, and mutant orbicules were smaller with reduced electron-dense sporopollenin, and the exine (including tectum, bacula, and nexine) of *osaldh2b* microspores was thinner than in WT. (C) At S10, the os*aldh2b* tapetum had become a cavity, with low-electron-density orbicules on its surface, indicating complete and premature tapetum degradation; the exine of *osaldh2b* microspores appeared thinner, with barren cytoplasm compared to WT. Ba, bacula; Ex, exine; Msp, microspores; Ne, nexine; Nu, nucleus; Or, orbicule; T, tapetum; Te, tectum. Scale bars: 1 µm.

We used transmission electron microscopy to study the developmental abnormalities at the microspore stages in more detail. At the early microspore stage (S9a), wild-type tapetal cytoplasm was highly condensed, nuclei were intact, and cells exhibited a prominent nucleolus (Fig. 8A, top). Strikingly, we observed no nucleolus in *osaldh2b* tapetum nuclei at this stage (Fig. 8A, bottom). At the middle microspore stage (S9b), the wild-type tapetum had collapsed and nuclei were lobed. Enlarged U-shaped orbicules were evident on the inner tapetal surface. The exine in wild-type microspores was well established with distinct nexine, tectum, and bacula layers (Fig. 8B, top). By contrast, at this stage in *osaldh2b* tapetal cells, nuclei appeared completely degenerated, orbicules were smaller, and electron-dense sporopollenin reduced. Moreover, the exine of *osaldh2b* microspores was much thinner compared with those in WT. The *osaldh2b* microspores contained few organelles in cytoplasm observed in electron-transparent channels (Fig. 8B, bottom). At the late microspore stage (S10) in wild-type, we observed further degenerated tapetum and vacuolated microspores with abundant cytoplasm (Fig. 8C, top). At this stage, *osaldh2b* tapetum exhibited cavities, with low-electron-density orbicules on its surface, indicating that its tapetum had completely and prematurely degraded. In addition, microspore exine was much thinner (Fig. 8C, bottom). We further investigated the expression of eight genes related to male reproductive development. The qRT-PCR results (Supplementary Fig. S5) showed that the mutation of *OsALDH2b* disrupted the expression of genes involved in tapetum degeneration (*TDR*, *UDT1*, *OsGAMYB*, *RTS*) and pollen wall formation (*WDA1*, *CYP704B2*, *CYP703A3*, *OsC6*).

**Fig. 9.**
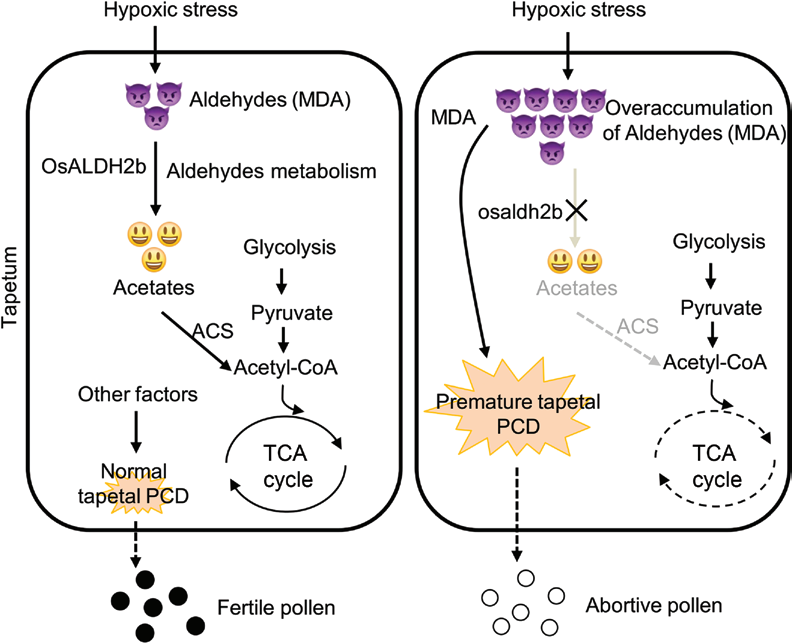
Model of OsALDH2b’s role in pollen development. Tapetal cells undergo high hypoxic stress at early developmental stages, triggering accumulation of reactive aldehydes (MDA). In wild-type rice, OsALDH2b converts aldehydes into acetates, which are incorporated into the TCA cycle, resulting in MDA homeostasis. Defective OsALDH2b causes excess aldehyde accumulation, which leads to premature tapetal PCD and cellular degradation, and pollen abortion. ACS, acetyl coenzyme A synthetase. TCA, tricarboxylic acid.

Together, these results suggested that defective mutation in *OsALDH2b* leads to excessive aldehyde accumulation, which causes early tapetal PCD, premature cellular degeneration, and aborted microspore development, resulting in male sterility (Fig. 9).

## Discussion

The tapetum is arguably the most important layer of anther tissue during male meiosis and microsporogenesis, providing enzymes, signals, and nutrients for pollen development via PCD-based cellular degeneration (Zhang *et al.*, 2011; Guo and Liu, 2012). Many components and factors participate in the process of tapetum development, such as transcription factors, receptor-like kinases, and transporters. The bHLH transcription factors TDR, EAT1/DTD, and TIP2 function as crucial positive regulators to promote tapetal PCD (Li *et al.*, 2006; Ji *et al.*, 2013; Niu *et al.*, 2013; Fu *et al.*, 2014). Mutation of their genes leads to vacuolated and prematurely degraded tapetum. In this study, we identified a rice male sterility mutant, *osaldh2b*; its wild-type gene encodes a conserved mitochondrial aldehyde dehydrogenase, OsALDH2b (Figs 1–4). Cytological analysis showed that *osaldh2b* exhibited more rapid, prominent degradation of tapetal cell nuclei and formation of abnormal tapetal secretory structures at microspore stages (Figs 7–8). Consistent with the nucleus degradation, tapetum DNA fragmentation (indicating PCD) occurred earlier in the *osaldh2b* mutant, at the prophase I stage (Fig. 6). Therefore we infer that OsALDH2b plays an important role in anther development and pollen formation by negatively regulating tapetal PCD. Additionally, expression analysis of some marker genes related to anther development indicates that the defective *OsALDH2b* caused disorder of the regulatory networks for anther development (Supplementary Fig. S5). The expression of *TDR* is increased from meiosis to microspore stages in *osaldh2b*. As the function of TDR is to promote the initiation of tapetal PCD (Li *et al.*, 2006), the up-regulated change of *TDR* expression is consistent with the earlier occurrence of tapetal PCD in *osaldh2b* anthers. Furthermore, the expression of *GAMYB*, which is involved in the down-regulation of *TDR* expression in anthers (Aya *et al.*, 2009; Liu *et al.*, 2010), is decreased in the mutant. According to the expression analysis, it seems that TDR may act downstream of OsALDH2b in regulating tapetal PCD, but this needs further investigation.

Dynamic redox status is an emerging factor affecting tapetum specification and timing degradation. Two Cys-rich metallothioneins, OsMT2b and OsMT-I-4b, have been identified as ROS scavengers. DTC1 interacts with OsMT2b and inhibits the ROS scavenging activity of OsMT2b to ensure timely production of ROS for proper initiation of tapetal PCD during early stage anther development (Yi *et al.*, 2016). On the contrary, OsMADS3 promotes the expression of *MT-I-4b* to eliminate the excess ROS during later stage anther development (Hu *et al.*, 2011). Previous studies have indicated that lipid peroxidation increases upon hypoxia in plants (Blokhina and Fagerstedt, 2010; Gupta *et al.*, 2017), resulting in production of reactive aldehydes including MDA; these are highly reactive with cellular compounds and nucleic acids (Voulgaridou *et al.*, 2011; Heymann *et al.*, 2018). ALDHs are major enzymes for selective elimination of aldehydes in animals and plants. In plants, the ALDH family includes mitochondrial ALDH (mtALDH) and cytosolic ALDH (ctALDH) subgroups based on their cellular location (Kirch *et al.*, 2004; Gao and Han, 2009; Zhou *et al.*, 2012). The rice genome harbors two mtALDHs, OsALDH2a and OsALDH2b (Gao and Han, 2009). Here we report that OsALDH2b functions to regulate the proper levels of aldehydes during redox stress in the developing tapetum. When OsALDH2b is dysfunctional, excess aldehydes accumulate in the tapetal cells (Fig. 5). Although the significant change of MDA accumulation slightly lags behind the observed early occurrence of tapetal PCD signal in *osaldh2b* (Figs 5, 6), we propose that the MDA accumulation might serve as a signal to initiate the premature tapetal PCD in this mutant.

Although *OsALDH2b* is constitutively expressed in vegetative organs, especially in leaf, the *osaldh2b* mutant does not show vegetative defects. We reason that there might be functional divergence of mtALDH orthologs as described in maize (Liu and Schnable, 2002). Two mtALDHs, RF2A and RF2B, have differential accumulation and distinct enzymatic activities with their substrates; RF2A, but not RF2B, accumulates to high levels in tapetal cells and is involved in male fertility (Cui *et al.*, 1996; Liu *et al.*, 2001). Based on previous phylogenetic analysis of plant ALDHs, OsALDH2b is more similar to maize RF2A and OsALDH2a is more similar to RF2B (Tsuji *et al.*, 2003). A possible role of OsALDH2a may be to eliminate acetaldehyde in vegetative tissues, so as to increase submergence tolerance (Nakazono *et al.*, 2000). Altogether, we conclude that mtALDHs have undergone functional specialization during evolution to accommodate endogenous or exogenous stresses in different developmental organs.

## Supplementary data

Supplementary data are available at *JXB* online.

Fig. S1. CRISPR/Cas9 *OsALDH2b* knockout.

Fig. S2. Sequence alignment of ALDH plant orthologs.

Fig. S3. OsALDH2b amino acid sequence.

Fig. S4. *OsALDH2b* expression profile based on RiceXPro.

Fig. S5. Expression analysis of eight genes related to anther development.

Table S1. Molecular markers used for fine mapping.

Table S2. Primers used for vector construction and expression analysis.

Table S3. Genetic analysis of *osaldh2b*.

eraa045_suppl_Supplementary_Tables_S1_S3_Figures_S1_S5
