# Supplementary material for: The mitochondrial aldehyde dehydrogenase OsALDH2b negatively regulates tapetum degeneration in rice
Source: J Exp Bot. 2020 Jan 28;71(9):2551–60. doi: 10.1093/jxb/eraa045 (PMC7210758; doi:10.1093/jxb/eraa045)
Supplement: eraa045_suppl_Supplementary_Tables_S1_S3_Figures_S1_S5 [file eraa045_suppl_supplementary_tables_s1_s3_figures_s1_s5.pdf]

Supplementary data

Table S1. Molecular markers used for fine mapping

Marker	Forward primer (5’ to 3’)	Reverse primer (5’ to 3’)
M1	GGTTGGTTCAATCACGCATC	GTCTTGCTCAGATCTGCCTT
M2	TCCTAGTTGCTTCCTGTG	ACTCAATGCATAAAGCTG
M3	TTTGCTGGCTACCTGCTC	TCTACATCATGTGATGC
M4	AGCTACGGTTTCCGAGTG	AATCGCGATAGGCATCG
M5	AATTCGGCTCCGGTCAC	TCTGAATCTTCACTGTCG
M6	AACCACACAAGTCCATC	TCAACCGTCATATATGTC
M7	TCTATTTGAATATCCAACG	ACAGCATCACAGACCTTC
M8	TGACCTGCTCCATCCATC	ACGTGGTACAGATTACAG
M9	ATTCTCGCTGCTCTTGGG	ATCAGGCGTACTGTGCTG

Table S2. Primers used for vector construction and expression analysis

Primer	Sequence (5' to 3')	Purpose
OsALDH2b-F1	ATTCATGGATCCACGCGTCATGGCTGCCGCTGCTGCAAGGAG	For construction of functional complementary vector
OsALDH2b -R1	TCGTTCAGATCACTGTCGACATAGCATAAATTGCG	
OsALDH2b-T5F2	ATAACGCAATTTATGCTATGTCGACAGTGATCTG	
OsALDH2b-T5R2	CAAGCTTGCATGCCTGCAGGTTGGATCTAGCAACGACTCCCTCG	
pOsALDH2b-T5F	GCTCGGTACCCGGGGATCCATCCTCACATGCATCGGCTGTACC	
pOsALDH2b-T5R	TGCAGCAGCGGCAGCCATGACCGATACCTCCCTCC	
OsALDH2b-RT-F	AATCTTATGGACGGATGCG	For qRT-PCR
OsALDH2b-RT-R	AGTAACATGAGCACCAACCAC	
OsActin1-F	CACATTCCAGCAGATGTGGA	
OsActin1-R	ACCACAGGTAGCAATAGGTA	
OsALDH2b-cF	ATGTCAAAGCTTCATGGCTGCCGCTGCTGCAAGGAG	For subcellular localization and prokaryotic expression
OsALDH2b-cR	TGTTACAAGCTTCAACCACGCGGCGTTCTTGATCG	
OsALDH2b-cFD	AGATTCGGATCCATGAGCACTGCAGCAGTAGCAGAGGAGC	
HPT-F	ATTTGTGTACGCCCGACAGT	For screening of transgenic plants
HPT-R	GTGCTTGACATTGGGGAGTT	
TDR-F	TGCTCTGGGAGCACAAGCC	For expression analysis of genes related to anther development
TDR-R	CTCGCTGTCCCTCACCATG	
UDT1-F	ACCAAGGTGCTGGAAGCACT	
UDT1-R	GTAGTCAGGAGTGTCTCAGAT	
OsGAMYB-F	GCGACGGTATCATGTTCAAT	
OsGAMYB-R	GTCGCATAAGAGAACATCTG	
WDA1-F	GTAGCGCAAGCATTATGCAA	
WDA1-R	CCTTGTCACCACATTCATGC	
CYP704B2-F	GCTGGTTGATGACTTCACCT	
CYP704B2-R	CGACAGTATGTCGTGCTTGAT	
CYP703A3-F	GAGTGCATCCCTTGATGATG	
CYP703A3-R	ACTCGTTGGTCACCGATGAT	
RTS-F	ACATGTGGACTCGCTTGACT	
RTS-R	CATGGCTGCATGCAGATTCAT	
OsC6-F	CTCCATCTGCCTGAGTATAT	
OsC6-R	GTCCATGCATGTTGCAGAAT	

Table S3. Genetic analysis of *osaldh2b*

Populations	WT (no.)	Mutant (no.)	χ^2 (3:1)	*P* value
Progenies of heterozygote (OsALDH2b/*osaldh2b*)	627	214	0.089	0.8 > *P* > 0.7
F_2 (*osaldh2b* × HHZ)	507	145	2.650	0.2 > *P* > 0.1

Notes: f = 1, χ^2 (3:1) < 3.84, *P* > 0.05.

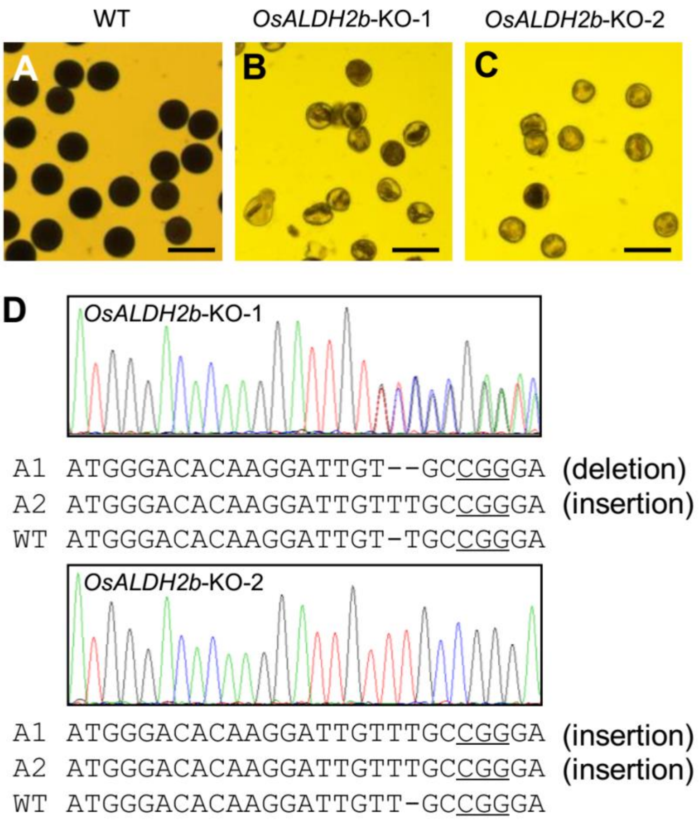

Fig. S1. CRISPR/Cas9 *OsALDH2b* knockout. (A-C) Pollen fertility of WT and two knockout lines (*OsALDH2b*-KO-1 and *OsALDH2b*-KO-2). (D) The genotyping of the target site in the knockout lines. A1, A2, mutant alleles of the lines.

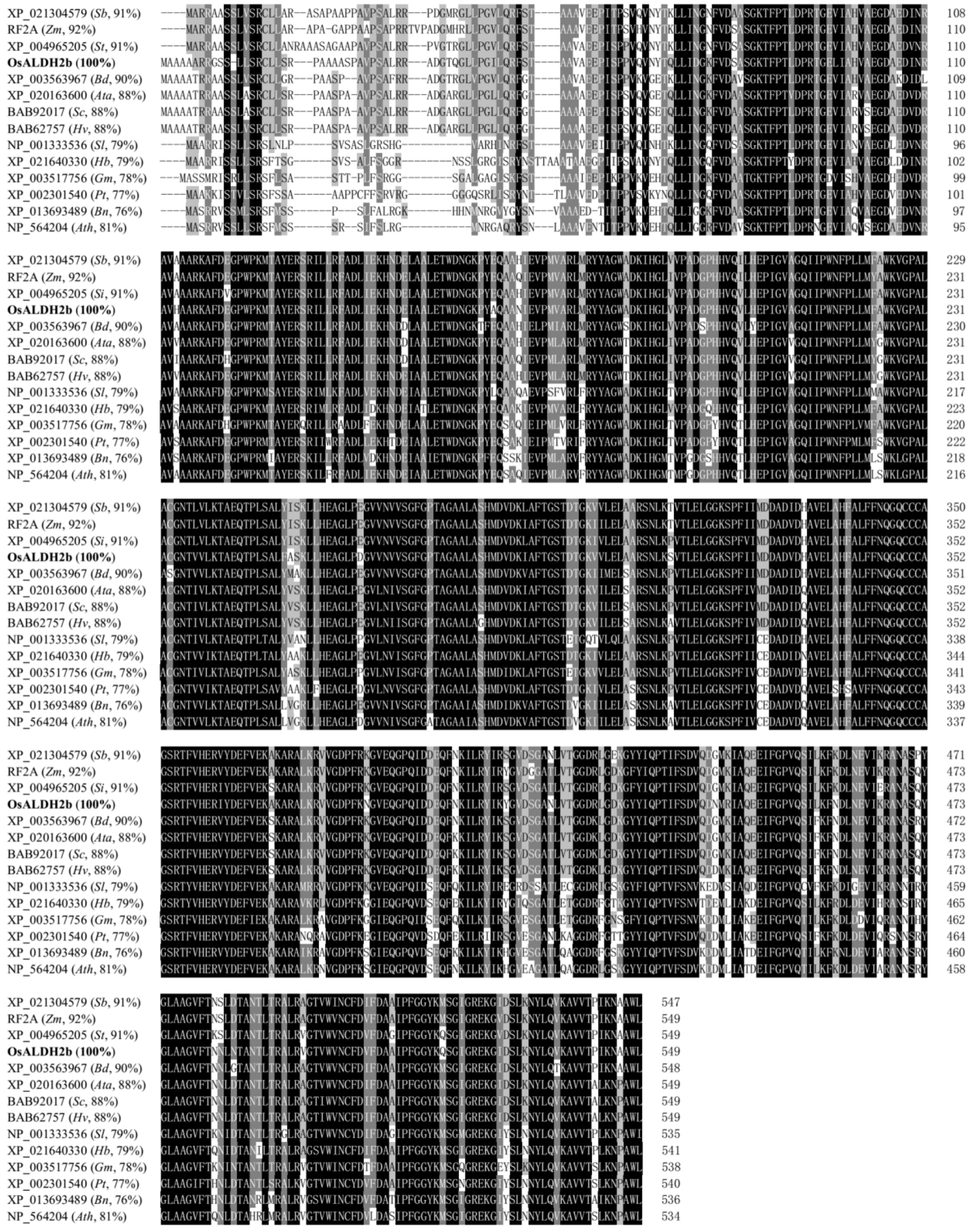

Fig. S2. Sequence alignment of ALDH plant orthologs. Similarities of the sequences with OsALDH2b were given after corresponding species names. *Sb, S. bicolor*; *Zm, Z. mays*; *Si, S. italica*; *Bd, B. distachyon*; *Ata, A. tauschii*; *Sc, S. cereal*; *Hv, H. vulgare*; *Sl, S. lycopersicum*; *Hb, H. brasiliensis*; *Gm, G. max*; *Pt, P. trichocarpa*; *Bn, B. napus*; *Ath, A. thaliana*.

```
  1 MAAAAARRGS SLLSRCLLSR PAAAASPAVP SALRRADGTQ GLLPGILQRF STAAVAEEPI
                                                ▼
 61 SPPVQVNYTQ LLIDGKFVDS ASGKTFPTLD PRTGELIAHV AEGDAEDINR AVHAARKAFD
121 EGPWPKMTAY ERSRILLRFA DLIEKHNDEI AALETWDNGK PYAQAANIEV PMVARLMRYY
181 AGWADKIHGL VVPADGPHHV QVLHEPIGVA GQIIPWNFPL LMFAWKVGPA LACGNTVVLK
241 TAEQTPLSAL FASKLLHEAG LPDGVVNVVS GFGPTAGAAL ASHMDVDKIA FTGSTDTGKV
301 VLELAARSNL KSVTLELGGK SPFIIMDDAD VDHAVELAHF ALFFNQGQCC CAGSRTFVHE
361 RIYDEFVEKA KARALKRVVG DPFKNGVEQG PQIDDEQFNK ILRYIKYGVD SGANLVTGGD
421 RLGDKGYYIQ PTIFSDVQDN MRIAQEEIFG PVQSILKFND LNEVIKRANA SQYGLAAGVF
481 TNNLNTANTL TRALRVGTVW VNCFDVFDAA IPFGGYKQSG IGREKGIDSL KNYLQVKAVV
541 TPIKNAAWL.
```

Fig. S3. OsALDH2b amino acid sequence. Putative mitochondrial targeting sequence is underlined. The arrowhead markers the start point of mutation in *osaldh2b*.

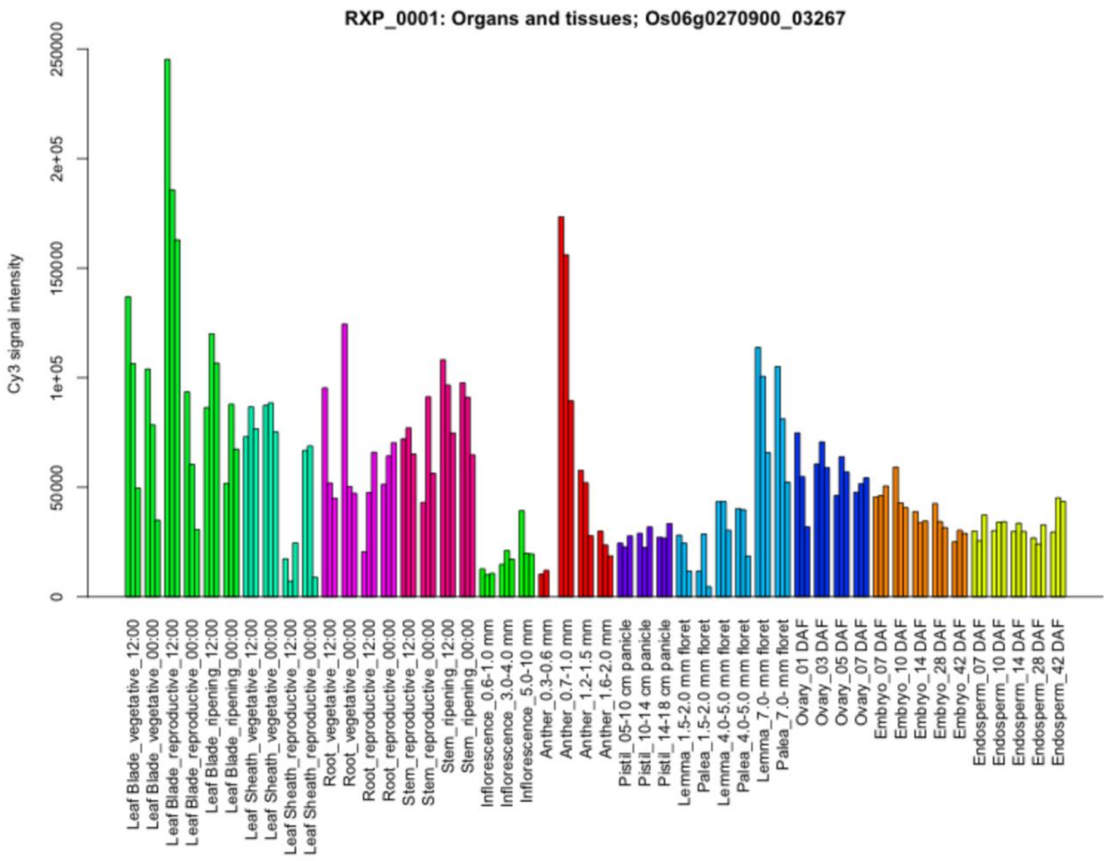

Fig. S4. *OsALDH2b* expression profile based on RiceXPro (http://ricexpro.dna.affrc.go.jp/)

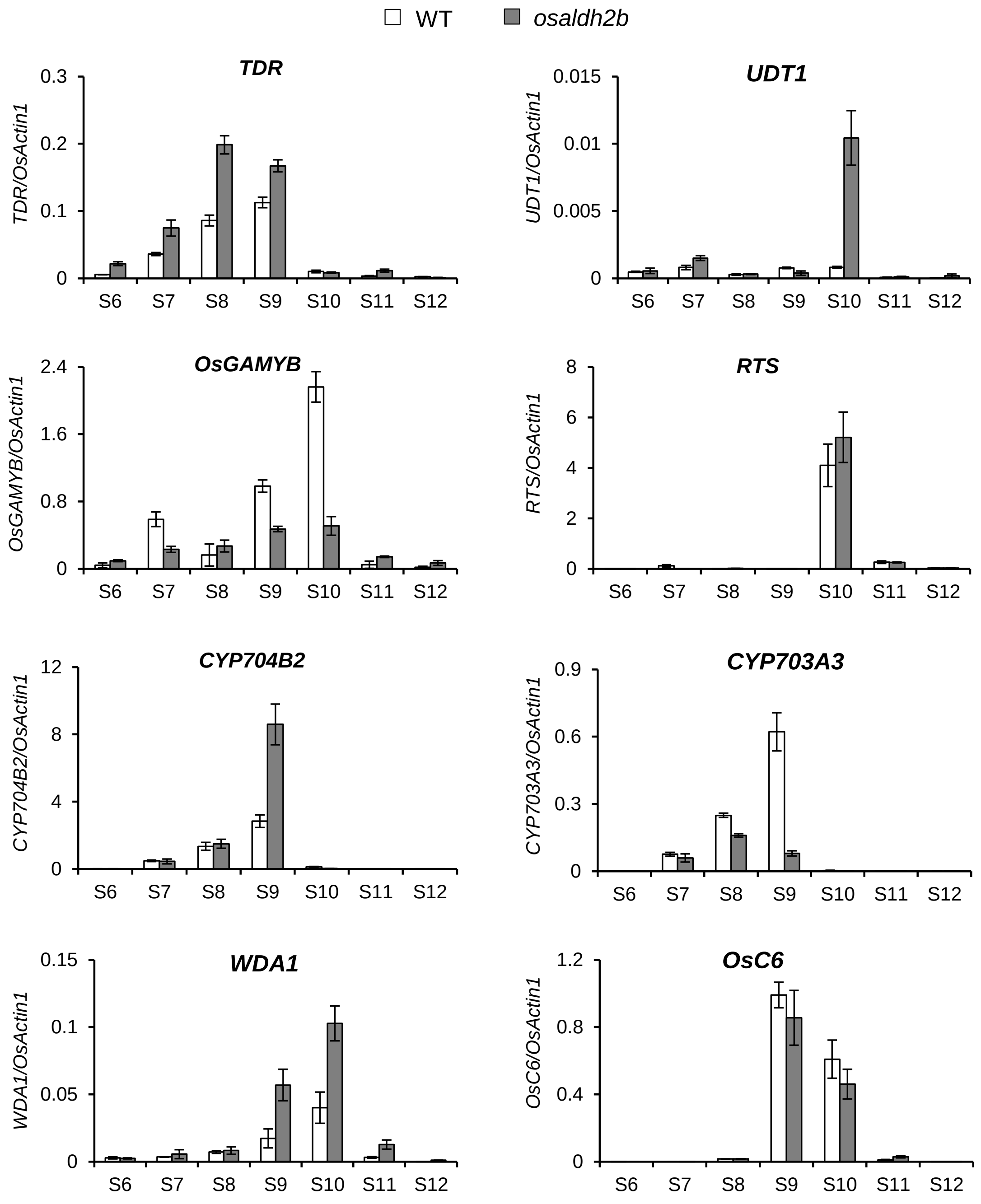

Fig. S5. Expression analysis of eight genes related to anther development. The relative expression levels indicate the ratios of target gene/*OsActin1*. S6-S12, anthers from stage 6 to stage 12. Data are shown as means ± SD (n = 3).
